# Optimization of Cellulose Nanofiber Loading and Processing Conditions during Melt Extrusion of Poly(3-hydroxybutyrate-*co*-3-hydroxyhexanoate) Bionanocomposites

**DOI:** 10.3390/polym15030671

**Published:** 2023-01-28

**Authors:** Siti Shazra Shazleen, Fatimah Athiyah Sabaruddin, Yoshito Ando, Hidayah Ariffin

**Affiliations:** 1 Department of Bioprocess Technology, Faculty of Biotechnology and Biomolecular Sciences, Universiti Putra Malaysia UPM, Serdang 43400, Selangor, Malaysia; 2 Graduate School of Life Science and Systems Engineering, Kyushu Institute of Technology, 2-4 Hibikino, Wakamatsu-ku, Kitakyushu-shi, Fukuoka 808-0196, Japan; 3 Laboratory of Biopolymer and Derivatives, Institute of Tropical Forestry and Forest Products, Universiti Putra Malaysia UPM, Serdang 43400, Selangor, Malaysia

**Keywords:** poly(3-hydroxybutyrate-*co*-3-hydroxyhexanoate), cellulose nanofiber, bionanocomposite, melt-extrusion processing, optimization, response surface methodology

## Abstract

This present study optimized the cellulose nanofiber (CNF) loading and melt processing conditions of poly(3-hydroxybutyrate-*co*-3-hydroxyhexanoate) P(HB-*co*-11% HHx) bionanocomposite fabrication in twin screw extruder by using the response surface methodology (RSM). A face-centered central composite design (CCD) was applied to statistically specify the important parameters, namely CNF loading (1–9 wt.%), rotational speed (20–60 rpm), and temperature (135–175 °C), on the mechanical properties of the P(HB-*co*-11% HHx) bionanocomposites. The developed model reveals that CNF loading and temperature were the dominating parameters that enhanced the mechanical properties of the P(HB-*co*-11% HHx)/CNF bionanocomposites. The optimal CNF loading, rotational speed, and temperature for P(HB-*co*-11% HHx) bionanocomposite fabrication were 1.5 wt.%, 20 rpm, and 160 °C, respectively. The predicted tensile strength, flexural strength, and flexural modulus for these optimum conditions were 22.96 MPa, 33.91 MPa, and 1.02 GPa, respectively, with maximum desirability of 0.929. P(HB-*co*-11% HHx)/CNF bionanocomposites exhibited improved tensile strength, flexural strength, and modulus by 17, 6, and 20%, respectively, as compared to the neat P(HB-*co*-11% HHx). While the crystallinity of P(HB-*co*-11% HHx)/CNF bionanocomposites increased by 17% under the optimal fabrication conditions, the thermal stability of the P(HB-*co*-11% HHx)/CNF bionanocomposites was not significantly different from neat P(HB-*co*-11% HHx).

## 1. Introduction

The growing use of plastics around the world has led to an increase in plastic waste. In Malaysia, plastic waste constituted 19% of the total waste generated where most of the commodity plastics are derived from petroleum, and they are single use, i.e., they are be discarded after being used only once [1]. This leads to the accumulation of disposal plastic waste that mostly ends up in landfills or dumps in the open environment. According to Jambeck et al. [2], Malaysia was placed eighth among the world’s top 10 countries for having poorly managed plastic waste. In light of the environmental damage caused by plastic waste pollution, and also the difficulties of managing that waste on land and in water, there is indeed an urgent need to establish sustainable and cost-effective solutions. Therefore, recent advancements in biodegradable and recyclable polymers are essential, considering the uncertainty of petroleum usage worldwide. Manufacturing industries are transitioning to more eco-friendly, sustainable economic production as a consequence of the intense pace of scientific and technological advancement. Today, the most well-known and widely used polymers in a multitude of areas are polylactic acid (PLA), polycaprolactone (PCL), polyglycolide (PGA), and polyhydroxyalkanoates (PHAs). Among all, PHA has drawn significant attention as one of the most viable substitutes for synthetic polymers. This is because PHAs are more compostable and biodegradable in marine conditions than PLA. Although PLA is compostable, it may remain in the ocean for up to 1000 years before it can be composted [3]. PCL and PGA are considered to be non-toxic, yet because of their higher crystallinity they degrade more slowly than PLA [4]. Moreover, PHA properties are comparable to most non-degradable materials [5], making PHA suitable for industrial uses, particularly in packaging.

PHA is recognized as a sustainable alternative among the most prominent synthesized and commercialized biodegradable polymers as it can be converted into water and carbon dioxide in the presence of oxygen, or into methane under anaerobic conditions without forming toxic products, by microorganisms found in water and soil [6]. PHAs are a type of linear biopolyester made up of hydroxyalkanoate (HA) units organized in a basic structure produced via bacterial fermentation and are currently being marketed as a means of creating a more sustainable future [7]. Their properties differ significantly depending on the structure and composition of their monomers [8]. PHAs offer several benefits over petroleum-based polymers, including the ability to be synthesized from renewable carbon sources, processability, and biodegradability. The most widespread and extensively studied member of this family is the homopolymer poly(3-hydroxybutyrate) (PHB) and the copolymers poly(3-hydroxybutyrate-*co*-3-hydroxyvalerate) (PHBV) and poly(3-hydroxybutyrate-*co*-3-hydroxyhexanoate) (PHBHHx).

PHBHHx is one of the most promising biodegradable aliphatic polyesters of the PHA family due to the fact that it has unique combination properties including full anaerobic degradability, moisture resistance, and good barrier properties [9]. PHBHHx has higher elastic characteristics and a wider processing window than PHB and PHBV due to the relatively long alkyl side chain, making it a suitable biopolymeric source for developing biocomposites with increased flexibility [10,11]. However, despite their potential, the effective utilization of PHBHHx-based materials is exacerbated by their low mechanical properties and difficulties in processing as compared to synthetic polymers [12]. In addition, PHBHHx-based materials are still hindered by their high production costs and are dependent on the performance of bacterial fermentation [13]. Their high manufacturing cost has surpassed the cost of manufacturing conventional plastics. These limitations have restrained the applicability of these materials in a wide range of applications. The incorporation of nanofillers, particularly bio-based nanofillers in PHBHHx, is seen as an ideal strategy for developing bio-based nanocomposites with superior mechanical properties.

Nanofillers have a higher aspect ratio than micro-sized fillers, giving them better reinforcement effects. Recent studies have focused on the use of nanocellulose, particularly cellulose nanofiber (CNF) as reinforcing bio-based nanomaterials. CNF has been known for its outstanding properties such as high mechanical properties and thermal stability, large specific surface area, renewability, biodegradability, and biocompatibility properties that can be produced by mechanical or chemical treatments which are advantageous for reinforced polymers [14]. CNF has a low coefficient of thermal expansion of 0.1 ppm/K; an estimated strength of 2–3 GPa, which is five times stronger than mild steel; and a high Young’s modulus of 130–150 GPa [15]. Recently, the effect of CNF as a reinforcement material for PHBHHx has been widely reported [14,15,16,17]. Most studies agreed that the addition of CNF can enhance the mechanical properties of PHBHHx bionanocomposites significantly.

Nonetheless, CNF loading beyond a certain percentage can be detrimental as it may lead to significant nanofiller agglomerations. Several studies have documented that the improvement of polymer nanocomposite may endure immense difficulty attributable to the dispersion of nanofibers [18,19,20,21,22]. The hypothesis is that if the nanofibers are evenly distributed throughout the polymer matrix, the optimal nanocomposite properties can be attained. It should be highlighted at this stage that proper alignment and control of nanofiber dispersion have remained a key problem for many years [23]. The effectiveness of load transfer between the nanofibers and the polymer matrix and subsequently the mechanical properties of nanocomposite are both governed by the strength of adhesion in the interface region [23]. Consequently, the characteristics of the nanocomposite deteriorate if there is inadequate adhesion at the interphase.

In complement to the CNF reinforcing effect, in practice, the applied melt-processing process and conditions can have a profound effect on the mechanical properties [12]. Nonetheless, studies on the mechanical properties of PHBHHx/CNF bionanocomposites under the influence of processing temperature and shear stress have been scarcely reported. To our knowledge, there are no studies that explicitly correlate variations in mechanical properties to the practical processing parameters and CNF loading used in melt extrusion of PHBHHx. Identifying the optimal values for these parameters to enhance the mechanical properties of the bionanocomposite is a challenging and complicated task as there are so many design parameters to consider. In light of this, the prediction and assessment of design parameters is vital for the optimum design of bionanocomposites for a particular application. However, few studies to date have quantitatively optimized the mechanical characteristics of PHBHHx/CNF bionanocomposites depending on the design parameters. Hence, the present in-depth research was performed with the purpose of improving the mechanical characteristics of P(HB-*co*-11% HHx) through the optimization of CNF loading and processing conditions. Through the use of Design-Expert software, mathematical models were generated between the aforementioned parameters and responses. The validation experiment was conducted to verify whether the obtained optimal conditions result in the intended mechanical properties for P(HB-*co*-11% HHx)/CNF bionanocomposites.

## 2. Materials and Methods

### 2.1. Materials

Poly(3-hydroxybutyrate-*co*-3-hydroxyhexanoate) with 11 mol% of HHx unit, P(HB-*co*-11% HHx) as determined by ^1^H-NMR was supplied by ©KANEKA Biodegradable Polymer ^TM^ (Kaneka Co., Osaka, Japan). Spray-dried CNF was purchased from ZoepNano Sdn. Bhd., (Serdang, Malaysia) and used in this experiment as nanofiller. The CNF powder had an average particle size of less than 100 nm.

### 2.2. P(HB-co-11% HHx) Bionanocomposite Fabrication and Molding

P(HB-*co*-11% HHx)/CNF bionanocomposite was fabricated by using twin-screw extruder (Labtech Engineering Co., Ltd., Bangkok, Thailand) with the screw diameter of 16 mm. Prior to mixing, P(HB-*co*-11% HHx) powder was dried in an oven at 60 °C for 24 h to remove moisture because it is very essential to minimize the hydrolytic degradation during the processing at high temperature [24]. The P(HB-*co*-11% HHx) and CNF powder were mechanically mixed before being fed into the extruder. After the extrusion, the obtained filament was granulated by a pelletizer (SHEER SGS 25–E4, MAAG Group manufactures, Zurich, Switzerland) and then molded into 11 × 11 cm film with thickness of 1 mm by direct compression molding using a hydraulic hot press at temperature of 160 °C and 110 kg.cm^−2^ pressure for 10 min. Cooling was then performed for 30 min under the same pressure.

### 2.3. Characterization of P(HB-co-11% HHx) Bionanocomposite

#### 2.3.1. Mechanical Analysis

Mechanical properties of bionanocomposites were analyzed using an Instron Universal Testing Machine–Instron 5566 (Instron, Norwood, MA, USA) with load cell of 10 kN at room temperature. Five dog-bone-shaped specimens for tensile strength were tested according to the standard method of ASTM D 638-05 with crosshead speed of 1 mm/min. Meanwhile, flexural strength and modulus tests were performed at 1.21 mm/min speed according to the standard method of ASTM D790. One-way ANOVA and Duncan’s multiple range test were used to statistically evaluate the mechanical properties of the fabricated bionanocomposites following the validation experiment.

#### 2.3.2. Experimental Design and Optimization

A face-centered central composite design (CCD), comprising three different factors, was used to run the experiment: CNF content (X_1_) (1 to 9 wt.%), rotational speed (X_2_) (20 to 60 rpm), and temperature (X_3_) (135 to 175 °C). In this study, CCD was used to determine the optimum melt-extrusion conditions in fabrication of P(HB-*co*-11% HHx)/CNF bionanocomposites with maximum mechanical properties. The temperature was set between 135 and 175 °C in consideration of the onset melting point of P(HB-*co*-11% HHx) from differential scanning calorimetry (DSC), which is approximately 155 °C, and onset degradation of CNF and P(HB-*co*-11% HHx) from thermogravimetric (TG) analysis around 280–290 °C. These variables were studied at five different levels coded as –α, −1, 0, +1, and +α, where α = 2. Actual and coded values of the variables are summarized in Table 1. The CCD consists of 19 runs including five replications of center points to determine pure error and reduce the variability in the data collection. The mechanical properties of the tensile strength (Y_1_), flexural strength (Y_2_), and flexural modulus (Y_3_) were recorded as responses. The experimental design arrangement was randomized to prevent systematic error and minimize the effects of uncontrolled factors.

The experimental data were analyzed, and response surface plots were generated using Design-Expert statistical software (Version 7.0, Stat-Ease Inc., Minneapolis, MN, USA). Analysis of variance (ANOVA) was used to determine the significance of each factor and the regression coefficient of the linear, quadratic, and interaction terms with a confidence level over 95% or a *p*-value lower than 0.05. The influence of the factors on the responses was illustrated using a contour plot, and the optimal levels were identified. Actual experimentation was performed to verify and validate the predicted optimal conditions obtained from software for CNF content, rotational speed, and temperature. Data were fitted to a second-order polynomial equation as shown in Equation (1), where Y_1_, Y_2_, and Y_3_ are the responses; X_1_, X_2_, and X_3_ are the varied factors ranging from –2 to 2, which influence the response Y; β_0_ is the constant coefficient; β_1_, β_2_, and β_3_ are linear coefficients; β_11_, β_22_, and β_33_ are quadratic coefficients; and β_12_, β_13_, and β_23_ are interaction coefficients. The validity and adequacy of the regression models were verified by comparing the experimentally obtained data with the fitted value predicted by the models.

Y = β_0_ + β_1_X_1_ + β_2_X_2_ + β_3_X_3_ + β_11_X_1_^2^ + β_22_X_2_^2^ + β_33_X_3_^2^ + β_12_X_1_X_2_ + β_13_X_1_X_3_ + β_23_X_2_X_3_ (1)

#### 2.3.3. Thermal Stability Analysis

Thermal stability of neat P(HB-*co*-11% HHx) and P(HB-*co*-11% HHx)/1.5% CNF bionanocomposites were analyzed using a thermogravimetric analyzer (TGA 4000, Perkin Elmer, Waltham, MA, USA). The samples weighing around 8–12 mg were placed on a ceramic pan and heated from 50 to 500 °C at a heating rate of 10 °C/min under nitrogen flow of 100 mL/min.

#### 2.3.4. X-ray Diffraction Analysis

X-ray diffraction (XRD) spectroscopy was used to quantify the crystallinity of bionanocomposites. An automated Shimadzu 6000 X-ray diffractometer (Tokyo, Japan) operating at 40 kV with a current of 20 mA and Cu radiation of =1.5406 between 2Ɵ = 10 and 50° was used for the experiment.

## 3. Results and Discussion

### 3.1. Response Surface Model Analysis

The design matrix generated by Design-Expert software included data on tensile strength, flexural strength, and flexural modulus allowed regression analysis to be performed to identify the best-fit model for the experimental data, with the derived regression equation being used to predict a particular response at points that are not included in regression [25]. The tensile strength, flexural strength, and flexural modulus were indicated to be correlated with CNF loading, rotating speed, and temperature by regression analysis of the experimental data. Different parameters including the model F value, the lack of fit F value, the coefficient of determination R^2^, adjusted R^2^, press value, and coefficient of variation (CV) were used to assess the model’s adequacy. The experimental and predicted values of responses are summarized in Table 2.

Full quadratic models were adopted as the best-fitting model for all responses as detailed in Table 3. The models were chosen using an ANOVA with a sufficient coefficient of determination (R2) (above 80%), insignificant lack-of-fit probability (*p* > 0.005), and significant model probability (*p* < 0.05). A significant *p*-value and an insignificant lack of fit, respectively, indicate a good model and a good fit of the model to the data [2,3]. The linear (X_1_, X_2_, X_3_), interactive (X_1_X_2_, X_1_X_3_, X_2_X_3_), and quadratic (X_1_^2^, X_2_^2^, X_3_^2^) *p*-values are presented in the same table. A lower *p*-value (*p* < 0.05) suggests that the corresponding coefficient is more significant.

The *p*-values for the lack-of-fit of tensile strength, flexural strength, and modulus were 0.1462, 0.6977, and 0.8991, respectively, which were higher than 0.05, signifying that the model had insignificant lack-of-fit. This is a good indicator that the proposed model fits the experimental data, and the factors have a significant effect on the responses. If the model exhibits significant lack-of-fit, it should not be applied to predict a particular response as it fails to represent data at points that were not included in the regression [25,26]. Ghelich et al. [27] mentioned that the significant lack-of-fit may be related to (i) replicate measurements with repetitive center point data that are consistent with each other, (ii) missing significant higher order non-standard terms or engagement in the model, (iii) larger residual errors compared to the pure error, or (iv) inadequate equal error at any points, i.e., heteroscedasticity (significant disparity between sizes of the observations), implying a more appropriate model fitting.

**Figure 1:**
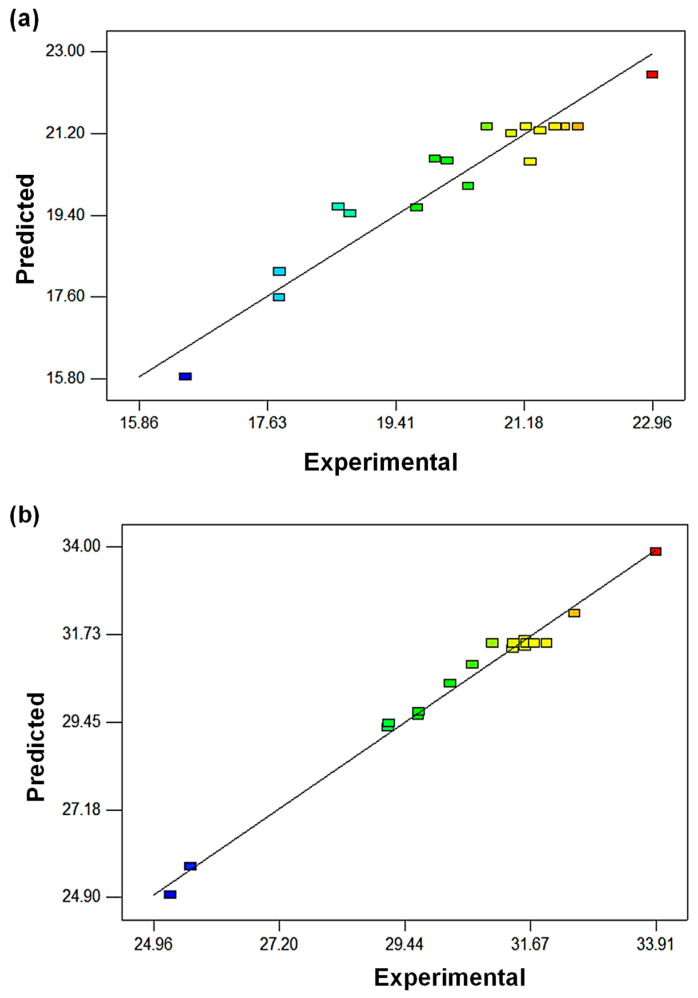

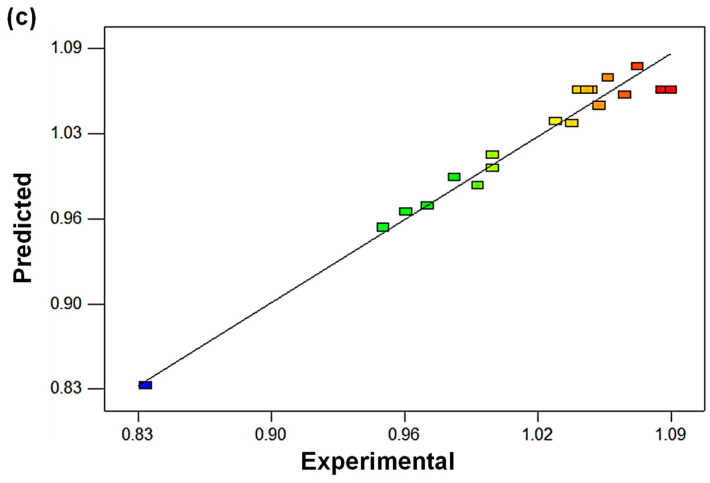
Experimental and predicted values for (**a**) tensile strength, (**b**) flexural strength, and (**c**) flexural modulus of P(HB-*co*-11% HHx)/CNF bionanocomposites.

The coefficient of determination R^2^ measures the quality of experimental data fitting to the model where the value was approximately near 1, highlighting that the dependent variable was predicted with less error than the independent variables of CNF loading, rotational speed, and temperature. The R^2^ values for tensile strength, flexural strength, and modulus were 0.9092, 0.9883, and 0.9619, respectively, signaling a high proportion of variability predicted by the models of 91%, 99%, and 96%, respectively, from CNF loading, rotational speed, and temperature of P(HB-*co*-11% HHx)/CNF bionanocomposite fabrication, as seen in Table 3. Moreover, a high R^2^ value close to 1 displays good agreement between predicted and reported results within the experimental range [28]. Figure 1 shows the plot of experimental by predicted values, where the proximity of the points scattered along the fitted line demonstrates agreement between experimental and predicted values, evidencing the adequacy of models to estimate the mechanical properties of P(HB-*co*-11% HHx)/CNF bionanocomposites prepared at varying CNF loadings, rotational speeds, and temperatures. Hence, these findings affirm that all the responses are affected by experimental factors.

The mathematical relationship between the response and variable process parameters can be established using response surface modeling. The final regression equations (in terms of coded factors) to predict the effect of factors on the responses are shown in Equations (2)–(4), where Y_1_, Y_2_, and Y_3_ represent tensile strength, flexural strength, and flexural modulus, respectively; X_1_, X_2_, and X_3_ are CNF loading, rotational speed, and temperature, respectively.

Y_1_ = 21.35 − 0.61 X_1_ − 0.17 X_2_ − 0.44 X_3_ − 0.020 X_1_^2^ − 0.11 X_2_^2^ − 1.15 X_3_^2^ + 0.12 X_1_X_2_ − 0.22 X_1_X_3_−1.15 X_2_X_3_ (2)

Y_2_ = 31.49 − 0.62 X_1_ − 0.23 X_2_ − 0.18 X_3_ + 0.29 X_1_^2^ + 0.077 X_2_^2^ − 1.54 X_3_^2^ + 0.045 X_1_X_2_
+ 0.43 X_1_X_3_ − 0.33 X_2_X_3_ (3)

Y_3_ = 1.06 + 0.014 X_1_ + 0.003375 X_2_ + 0.038 X_3_ − 0.007963 X_1_^2^ − 0.004713 X_2_^2^ − 0.037 X_3_^2^ + 0.0085 X_1_X_2_ + 0.012 X_1_X_3_ − 0.007963 X_2_X_3_ (4)

The polynomial equation in terms of uncoded variables of factors was obtained by exchange of coded variables with actual values as shown in Equations (5)–(7).

Y_1_ = −292.88125 + 1.20304 (CNF content) + 0.95185 (Rotational speed) + 3.82790 (Temperature) − 0.00499155 (CNF content)^2^ − 0.00106216 (Rotational speed)^2^
− 0.011550 (Temperature)^2^ + 0.006125 (CNF content)(Rotational speed) − 0.011 (CNF content)(Temperature) − 0.0059 (Rotational speed)(Temperature)(5)

Y_2_ = − 333.88437 − 4.40841 (CNF content) + 0.41136 (Rotational speed) + 4.78479 (Temperature) + 0.071529 (CNF content)^2^ + 0.000773649 (Rotational speed)^2^ −0.015414 (Temperature)^2^ + 0.00225 (CNF content)(Rotational speed) + 0.021250 (CNF content)(Temperature) − 0.00327 (Rotational speed)(Temperature) (6)

Y_3_ = − 8.61259 − 0.079280 (CNF content) + 0.013608 (Rotational speed) + 0.11970 (Temperature) − 0.0019907 (CNF content)^2^ − 0.0000471284 (Rotational speed)^2^ − 0.000373378 (Temperature)^2^ + 0.000425 (CNF content)(Rotational speed) + 0.000575 (CNF content)(Temperature) − 0.000075 (Rotational speed)(Temperature)(7)

### 3.2. Effect of Melt-Extrusion Processing Conditions on Mechanical Properties of P(Hb-Co-11% HHx)/CNF Bionanocomposites

**Figure 2:**
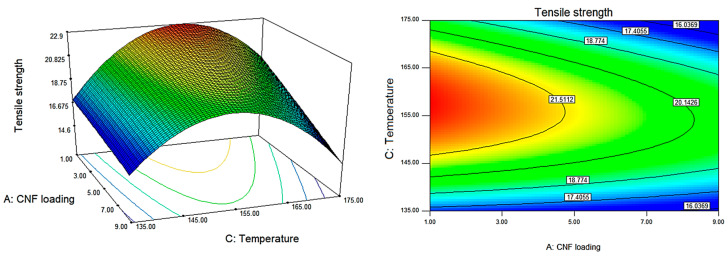
The 3D and response surface contour plots for the dependence of P(HB-*co*-11% HHx)/CNF bionanocomposite’s tensile strength on CNF loading and temperature as significant factors.

**Figure 3:**
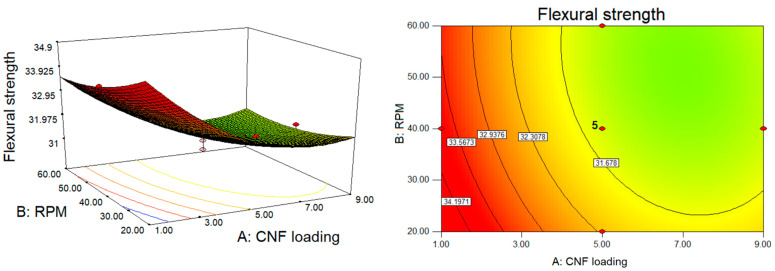
The 3D and response surface contour plots for the dependence of P(HB-*co*-11% HHx)/CNF bionanocomposite’s flexural strength on CNF loading and rotational speed as significant factors.

**Figure 4:**
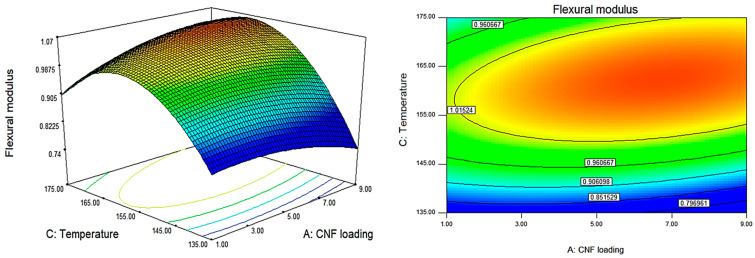
The 3D and response surface contour plots for the dependence of P(HB-*co*-11% HHx)/CNF bionanocomposite’s flexural modulus on CNF loading and temperature as significant factors.

The effect of each processing factor, namely CNF loading, rotating speed, and temperature, on the mechanical characteristics of P(HB-*co*-11% HHx)/CNF bionanocomposite was assessed using a quadratic regression model. The contour and response surface plots generated from the empirical predicted model in Equations (2)–)4) can be used to better assess the whole relationship between the independent variable X and the response variable Y, as depicted in Figure 2, Figure 3 and Figure 4. The response surface plots, which are shown from the pairwise combination of targeted variables by keeping other variables at their center point level, demonstrate the mutual interaction of the independent factors. The response surface plots were converted into a three-dimensional (3D) diagram to determine the optimal conditions for each variable towards maximum mechanical properties. Thus, in all response surface and 3D contour plots, the fixed variable is held at a rotational speed of 30 rpm.

Figure 2 depicts the 3D and response surface contour plots for the effects of CNF loading and temperature on tensile strength based on Equation (1). Results indicate that these variables are the most important factors influencing tensile strength (Table 3). It was observed that further increment in CNF loading of more than 3 wt.% reduced the tensile strength of the bionanocomposites. This might be due to the agglomeration of the CNF within P(HB-*co*-11% HHx) matrix which disrupted the compactness and the spherulite structure of the bionanocomposites [29,30]. Meanwhile, the tensile strength of P(HB-*co*-11% HHx)/CNF bionanocomposites increased as temperature rose from 135 to 155 °C before declining from 155 to 175 °C. This result may be explained by the fact that P(HB-*co*-11% HHx) twin-screw extrusion at high temperature can result in a reduction in molecular weight owing to thermal degradation [10,24]. It has been discovered that random chain scission is the process of degradation causing PHAs to rapidly lose molecular weight when exposed to heat [31].

Figure 3 the depicts 3D and response surface contour plots for the effects of CNF loading and rotational speed on flexural strength based on Equation (3). CNF loading (linear and quadratic) had a significant effect (*p* < 0.05) on flexural strength, as well the linear effect of rotational speed, interaction effect of CNF loading-rotational speed, and CNF loading-temperature and also the quadratic effect of temperature (Table 3). In comparison to the quadratic effect of temperature, the quadratic effect of CNF loading was less pronounced, with the coefficient of each factor of 1.54 and 0.29, respectively (Equation (3)). It was discovered that CNF dispersion in P(HB-*co*-11% HHx) was unaffected by rotating speed, as demonstrated by insignificant changes in tensile strength and flexural modulus. Conversely, a significant linear effect was noticed for flexural strength (Table 3), where a minor improvement could be noticed while processing P(HB-*co*-11% HHx) bionanocomposite at a slower rotational speed (Figure 3). Flexural strength decreases with the increase in CNF loading and rotational speed from 1 to 9 wt.% and 20 to 60 rpm, respectively.

Similar to flexural strength, significant quadratic effects of both CNF loading and temperature were observed against flexural modulus with no significant interactions between all factors (Table 3). Flexural modulus decreased with the increase in processing temperature from 155 to 175 °C, which is similar to the results of tensile and flexural strength. As aforementioned, fabricating bionanocomposites at high processing temperature leads to polymer degradation due to random chain scission, resulting in brittleness of the bionanocomposites [32,33]. Since the polymer molecular weight substantially decreases at temperatures above 155 °C, processing at those temperatures seems unsuitable. However, different from tensile and flexural strength, the addition of CNF up to 9 wt.% did not negatively affect the flexural modulus (Figure 4). In this study, CNF distribution in P(HB-*co*-11% HHx) was found to be unaffected by rotational speed, as proved by in Table 3, where no significant interaction can be seen between CNF loading and screw speed.

### 3.3. Response Surface Optimization of P(HB-co-11% HHx)/CNF Bionanocomposites

Numerical optimization was performed in accordance with the design and analysis, taking each criterion into consideration (Table 4). Due the severity of the effect on tensile and flexural strength as well as flexural modulus, these responses were set at a maximum value. As shown in Table 4, the optimal CNF loading, rotational speed, and temperature for P(HB-*co*-11% HHx)/CNF bionanocomposite fabrication were 1.5 wt.% CNF, 20 rpm, and 160 °C, respectively. For these ideal conditions, it was predicted that the tensile strength, flexural strength, and modulus would be 22.96 MPa, 33.91 MPa, and 1.022 GPa, respectively, with a maximum desirability of 0.929.

### 3.4. Validation Experiment

The mechanical properties of the P(HB-*co*-11% HHx)/CNF bionanocomposite fabricated at the proposed parameter were consistent with the predicted value throughout the validation experiment as tabulated in Table 5, with the exception of the tensile strength, which was 9% higher. This finding was very good and favorable in light of the objective of this study, which was to attain high mechanical properties.

The mechanical properties of the neat P(HB-*co*-11% HHx) and P(HB-*co*-11% HHx)/CNF bionanocomposites prepared under these ideal conditions are shown in Table 6. This result demonstrated the ability of CNF to increase tensile strength, flexural strength, and flexural modulus by 17, 6, and 20%, respectively.

### 3.5. Effect of CNF on Thermal Stability and Crystallinity Properties of P(HB-co-11% HHx)/CNF Bionanocomposites

**Figure 5:**
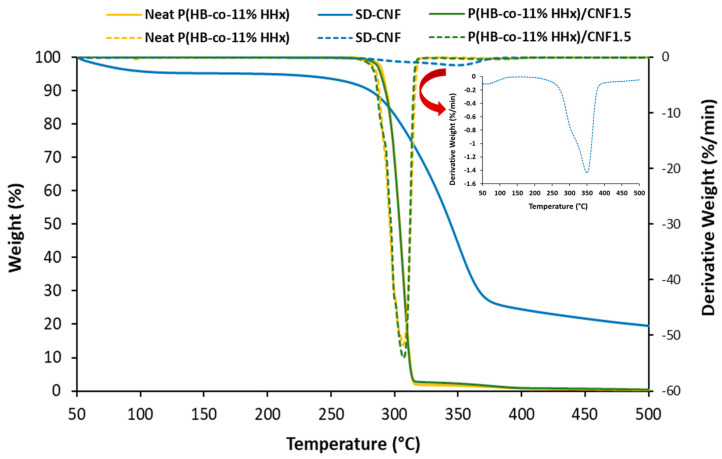
TG and DTG curves for neat P(HB-*co*-11% HHx) and P(HB-*co*-11% HHx)/CNF1.5 bionanocomposites (Red arrow shows the zoom in peak for SD-CNF for DTG).

The thermal stability of spray dried-CNF, neat P(HB-*co*-11% HHx) and P(HB-*co*-11% HHx)/CNF1.5 bionanocomposites was investigated by thermogravimetric analysis. The TG and DTG curves are presented in Figure 5, and the thermal degradation data are summarized in Table 7. T_10_ represents the temperature at which 10% of mass reduction was recorded, while T_max_ represents the temperature at maximum mass loss, which was taken from the DTG thermogram.

P(HB-*co*-11% HHx) thermal decomposition commenced at 280 °C and was completed at 320°C with single step degradation profile. As seen from Table 7, the T_10_ and T_max_ of neat P(HB-*co*-11% HHx) were 294 and 307 °C, respectively. Spray dried-CNF was less thermally stable at the beginning, where some weight loss started to occur at temperature around 100 °C, indicating the removal of moisture. Nevertheless, the T_max_ value was higher compared to P(HB-*co*-11% HHx). The addition of 1.5 wt.% CNF did not change the thermal stability of neat P(HB-*co*-11% HHx).

**Figure 6:**
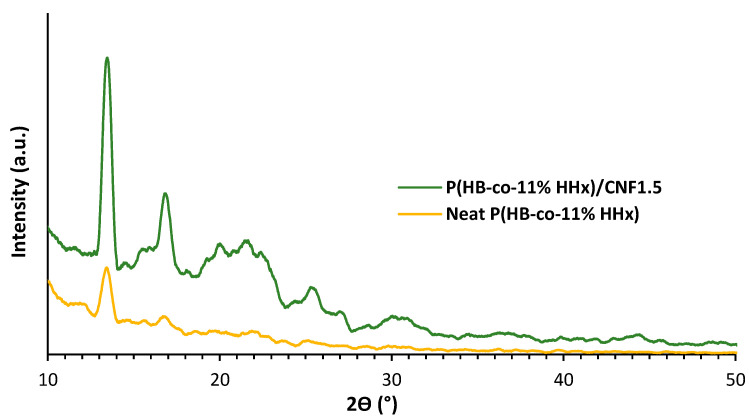
XRD patterns of neat P(HB-*co*-11% HHx) and optimized P(HB-*co*-11% HHx)/CNF1.5 bionanocomposites.

X-ray diffraction (XRD) analysis was performed to determine the crystallinity properties of neat P(HB-*co*-11% HHx) and optimized P(HB-*co*-11% HHx)/CNF1.5 bionanocomposites. Figure 6 displays the XRD patterns for both bionanocomposite samples, and the crystallinity index calculated is tabulated in Table 8.

Sharp crystal peaks are visible in this sample, and their intensity was noticeably higher than that seen in the neat P(HB-*co*-11% HHx) sample, in accordance with the pattern of a bionanocomposite film containing 1.5 wt.% of CNF. This can be linked to the effective CNF distribution in the film matrix. This finding demonstrated that the addition of CNF promotes the growth of crystalline areas in the P(HB-*co*-11% HHx) matrix as evidenced by the increase in crystallinity index from 25.1 to 30.1%. As proved in Table 6, it has been suggested that an increase in crystallinity leads to an increase in the strength and modulus of bionanocomposites. Additionally, there is no peak shift in the XRD pattern of P(HB-*co*-11% HHx)/CNF bionanocomposites, indicating that the melt compounding by the twin screw extrusion process did not alter their crystal structures [24].

## 4. Conclusions

P(HB-*co*-11% HHx)/CNF bionanocomposites fabricated using twin screw extruders were evaluated and predicted, and their melt-extrusion processing parameters were optimized using response surface modeling analysis based on the CCD method. The individual and interaction effects of three important melt-extrusion processing conditions, namely CNF loading, rotation speed, and temperature, on mechanical properties of P(HB-*co*-11% HHx)/CNF bionanocomposites (tensile strength, flexural strength, and modulus) were adequately modeled and optimized. It was discovered that CNF loading and temperature have a substantial effect on the mechanical properties of the P(HB-*co*-11% HHx)/CNF bionanocomposites; however, rotational speed has a less influential effect on mechanical properties except flexural modulus. At optimum CNF loading, rotational speed, and temperature of 1.5 wt.%, 20 rpm, and 160 °C, tensile strength, flexural strength, and flexural modulus are reported to be 22.96 MPa, 33.91 MPa, and 1.022 GPa, respectively. The response values obtained from the validation experiment were in good agreement with the predicted values. Validation tests proved that the response surface equations were adequate for predicting responses. The results of the TG and DTG study indicated that the thermal stability of the P(HB-*co*-11% HHx) matrix did not differ significantly from neat P(HB-*co*-11% HHx) when the optimum amount of CNF was introduced. The XRD analysis revealed that the addition of CNF increased crystallinity, which promotes the formation of crystalline regions in the P(HB-*co*-11% HHx) matrix. This research highlights the importance of optimal melt-extrusion processing conditions and their influence on the mechanical properties of P(HB-*co*-11% HHx)/CNF bionanocomposites. The findings of this research are anticipated to aid in the invention of novel P(HB-*co*-11% HHx) materials for packaging and other applications, taking into consideration the relevance of the correlation between melt-extrusion process parameters and the potential for process optimization to enhance the mechanical properties of P(HB-*co*-11% HHx). This process optimization can be further used as one of the technical developments for bio-based nanocomposites derived from green materials with excellent prospects in novel high performance food packaging materials. With the optimum process conditions, the properties such as high mechanical performance with additional desired function such good thermal and barrier properties can be achieved. In addition, the application of bio-based materials to replace the synthetic fossil-based materials provides a range of benefits for various economic entities. Positive feedback has also been reported concerning ecological, economic, and social aspects associated with the transition from traditional plastic to bio-based plastics that can be widely used as packaging materials and act as a significant driver of the industry’s growth.

## Figures and Tables

**Table 1:** Central composite design matrix of coded and actual factor level.

Run	CNF Loading (wt.%), X_1_		Rotational Speed (rpm), X_2_		Temperature (°C), X_3_	
	Coded	Actual	Coded	Actual	Coded	Actual
1	0	5	2	60	0	155
2	0	5	0	40	0	155
3	0	5	0	40	0	155
4	–2	1	0	40	0	155
5	–1	3	–1	30	1	165
6	0	5	0	40	0	155
7	0	5	0	40	0	155
8	1	7	–1	30	1	165
9	0	5	0	40	2	175
10	0	5	–2	20	0	155
11	0	5	0	40	0	155
12	–1	3	1	50	1	165
13	–1	3	–1	30	–1	145
14	2	9	0	40	0	155
15	1	7	1	50	1	165
16	1	7	1	50	–1	145
17	–1	3	1	50	–1	145
18	1	7	–1	30	–1	145
19	0	5	0	40	–2	135

**Table 2:** The experimental and predicted values of responses.

Run	CNF Loading (wt.%), X_1_		Rotational Speed (rpm), X_2_		Temperature (°C), X_3_	
	* Exp.	** Pred.	* Exp.	** Pred.	* Exp.	** Pred.
1	21.27	20.58	31.36	31.34	1.05	1.05
2	21.63	21.35	31.74	31.49	1.09	1.06
3	21.73	21.35	31.61	31.49	1.05	1.06
4	22.96	22.49	33.91	33.87	1.00	1.00
5	21.21	21.35	30.64	30.93	1.03	1.03
6	21.93	21.35	31.96	31.49	1.05	1.06
7	21.61	21.35	31.00	31.49	1.04	1.06
8	18.78	19.43	30.25	30.45	1.06	1.07
9	16.50	15.86	25.26	24.96	0.99	0.99
10	21.41	21.26	32.46	32.26	1.04	1.03
11	20.67	21.35	31.37	31.49	1.08	1.06
12	18.61	19.58	29.68	29.72	1.00	1.01
13	20.12	20.60	31.55	31.49	0.96	0.97
14	20.41	20.04	31.58	31.40	1.06	1.05
15	17.80	18.16	29.15	29.43	1.07	1.08
16	19.95	20.65	29.67	29.60	0.98	0.98
17	21.01	21.19	31.58	31.59	0.97	0.97
18	19.70	19.56	29.14	29.31	0.95	0.95
19	17.79	17.60	25.61	25.69	0.84	0.83

* Exp.: experimental; ** Pred.: predicted.

**Table 3:** Analysis of variance (ANOVA) for response surface quadratic model.

	Tensile Strength (MPa), Y_1_	Flexural Strength (MPa), Y_2_	Flexural Modulus (GPa), Y_3_
Model	0.0010 *	<0.0001 *	<0.0001 *
Linear	–	–	–
X_1_–CNF content	0.0078 *	<0.0001 *	0.0085 *
X_2_–Rotational speed	0.3705	0.0197 *	0.4362
X_3_–Temperature	0.0391 *	0.0523	<0.0001 *
Interaction	–	–	–
X_1_X_2_	0.6425	0.7058	0.1807
X_1_X_3_	0.4108	0.0051 *	0.0812
X_2_X_3_	0.0460 *	0.0195 *	0.2325
Quadratic	–	–	–
X_1_^2^	0.8958	0.0021 *	0.0441 *
X_2_^2^	0.4919	0.2788	0.1997
X_3_^2^	<0.0001 *	<0.0001 *	<0.0001 *
Lack of fit	0.1462 **	0.6977 **	0.8991 **
R^2^	0.9092	0.9883	0.9619
Standard deviation	0.72	0.33	0.017

* Statistically significant at *p* < 0.05 for model; ** statistically insignificant at *p* > 0.05 for the lack of fit.

**Table 4:** The settings and solutions of the numerical optimization criterion.

Factor Constraints							
Name	Goal		Lower Limit		Upper Limit		
X_1_	Is in range		1.00		9.00		
X_2_	Is in range		20.00		60.00		
X_3_	Is in range		135.00		175.00		
**Response Constraints**							
Y_1_	Maximize		16.50		22.96		
Y_2_	Maximize		25.26		33.91		
Y_3_	Maximize		0.836		1.086		
**Optimum Solutions**							
Number	X_1_	X_2_	X_3_	Y_1_	Y_2_	Y_3_	Desirability
1	1.54	20.00	159.53	22.96	33.91	1.022	0.929
2	1.54	20.00	159.33	22.96	33.94	1.022	0.928
3	1.50	20.67	159.40	22.96	33.91	1.022	0.928
4	1.46	21.28	158.84	22.96	33.99	1.021	0.927

**Table 5:** Comparison between predicted and experimental values of P(HB-*co*-11% HHx)/CNF bionanocomposites fabricated at optimal conditions.

	Predicted	Experimental
Tensile strength (MPa), Y_1_	22.16	25.11 ± 0.4
Flexural strength (MPa), Y_2_	33.91	32.46 ± 0.4
Flexural modulus (GPa), Y_3_	1.02	1.00 ± 0.1

**Table 6:** Mechanical properties of neat P(HB-*co*-11% HHx) and P(HB-co-11% HHx)/CNF1.5 bionanocomposites.

	Neat P(HB-*co*-11% HHx)	P(HB-*co*-11% HHx)/CNF1.5
Tensile strength (MPa)	21.48 ± 0.4 ^b^	25.11 ± 0.4 ^a^
Flexural strength (MPa)	30.54 ± 0.7 ^b^	32.46 ± 0.4 ^a^
Flexural modulus (GPa)	0.83 ± 0.1 ^b^	1.00 ± 0.1 ^a^

All data are means of five replicates ± S.D. The superscript letters indicate significant difference (*p* < 0.05) according to Duncan’s multiple range test.

**Table 7:** Thermal degradation temperatures of neat P(HB-*co*-11% HHx) and P(HB-*co*-11% HHx)/CNF at 10 wt.% of weight loss (T_10_) and maximum degradation temperature (T_max_).

Sample	T_10_ (°C)	T_max_ (°C)
Neat P(HB-*co*-11% HHx)	294	307
SD–CNF	281	350
Optimized P(HB-*co*-11% HHx)/CNF1.5	293	307

**Table 8:** Crystallinity index (CI) of neat P(HB-*co*-11% HHx) and P(HB-*co*-11% HHx)/CNF bionanocomposites.

Sample	Crystallinity Index (%)
Neat P(HB-*co*-11% HHx)	25.1
P(HB-*co*-11% HHx)/CNF1.5	30.1

## Data Availability

Not applicable.
